# Supplementary material for: Lowered Quality of Life in Long COVID Is Predicted by Affective Symptoms, Chronic Fatigue Syndrome, Inflammation and Neuroimmunotoxic Pathways
Source: Int J Environ Res Public Health. 2022 Aug 19;19(16):10362. doi: 10.3390/ijerph191610362 (PMC9408685; doi:10.3390/ijerph191610362)
Supplement: Supplementary file 1 [file ijerph-19-10362-s001.zip › ijerph-1860033-supplementary.pdf]

ELECTRONIC SUPPLEMENTARY FILE (ESF)

Lowered Quality of Life in Long COVID is Predicted by Affective Symptoms, Chronic Fatigue Syndrome, inflammation and Neuroimmunotoxic Pathways

(a,b,c,d) Michael Maes *, (e) Haneen Tahseen Al-Rubaye, (a,f) Abbas F. Almulla, (g) Dhurgham Shihab Al-Hadrawi, (b,c) Kristina Stoyanova, (h) Marta Kubera, (i) Hussein Kadhem Al-Hakeim

(a) Department of Psychiatry, Faculty of Medicine, Chulalongkorn University, Bangkok, Thailand
(b) Department of Psychiatry, Medical University of Plovdiv, Plovdiv, Bulgaria
(c) Research Institute, Medical University Plovdiv, Plovdiv, Bulgaria

(d) Deakin University, IMPACT, the Institute for Mental and Physical Health and Clinical Translation, School of Medicine, Barwon Health, Geelong, Australia
(e) College of Medical laboratory Techniques, Imam Ja'afar Al-Sadiq University, Najaf, Iraq
(f) Medical Laboratory Technology Department, College of Medical Technology, The Islamic University, Najaf, Iraq
(g) Al-Najaf Center for Cardiac Surgery and Transcatheter Therapy, Najaf, Iraq
(h) Laboratory of Immunoendocrinology, Department of Experimental Neuroendocrinology, Maj Institute of Pharmacology, Polish Academy of Sciences, 12 Smetna St., 31-343 Krakow, Poland
(i) Department of Chemistry, College of Science, University of Kufa, Iraq

Table S1. Socio-demographic data of healthy controls (HC) and Long COVID patients.

Variables	HC (n=39)	Long COVID (n=86)	F/X^2	df	p
Age (Years)	28.3 (7.6)	28.4 (6.2)	0.00	1/123	0.967
Sex (Female/Male)	15/24	24/62	1.39	1	0.238
Marital state (Single/Married)	17/22	37/49	0.00	1	0.953
Smoking (No/Yes)	26/13	59/27	0.05	1	0.830
Residency (Rural/urban)	8/31	15/71	0.17	1	0.681
Vaccination (A/Pf/S)	9/21/9	20/48/18	0.08	2	0.963
BMI (Kg/m^2)	25.60 (3.98)	26.15 (4.55)	0.42	1/123	0.517
Education (Years)	15.0 (1.3)	15.7 (1.7)	5.10	1/123	0.026

Results are shown as mean (SD): F: results of analysis of variance; X^2: analysis of contingency tables. Body Mass Index, A: AstraZeneca, Pf: Pfizer, S: Sinopharm
